# Role of MicroRNA in Response to Ionizing Radiations: Evidences and Potential Impact on Clinical Practice for Radiotherapy

**DOI:** 10.3390/molecules19045379

**Published:** 2014-04-24

**Authors:** Francesco Cellini, Alessio G. Morganti, Domenico Genovesi, Nicola Silvestris, Vincenzo Valentini

**Affiliations:** 1 Radiation Oncology Department, Policlinico Universitario Campus Bio-Medico; Via Álvaro del Portillo 200, 00144 Rome, Italy; 2 Radiotherapy Department, Università Cattolica del Sacro Cuore; Fondazione di Ricerca e Cura “Giovanni Paolo II”, Largo Agostino Gemelli 1, 86100 Campobasso, Italy; E-Mail: amorganti@rm.unicatt.it; 3 Radiation Oncology Department, Università Cattolica del Sacro Cuore; L.go Francesco Vito 1, 00168 Roma, Italy; E-Mail: vvalentini@rm.unicatt.it; 4 Radiation Oncology Department, Università “G. D'Annunzio”; Via dei Vestini 31, 66100 Chieti, Italy; E-Mail: d.genovesi@unich.it; 5 Medical Oncology Unit - Cancer Institute “Giovanni Paolo II”; Viale Orazio Flacco, 65, 70124 Bari, Italy; E-Mail: n.silvestris@oncologico.bari.it

**Keywords:** miRNA, miRNAs, microRNA, radiation, radiotherapy, resistance, review, tumor, DNA damage repair, radiosensibilization

## Abstract

MicroRNAs (miRNA) are small, non-coding, RNAs with gene expression regulator roles. As an important class of regulators of many cellular pathways, miRNAs are involved in many signaling pathways and DNA damage repair processes, affecting cellular radiosensitivity. Their role has led to interest in oncological implications to improve treatment results. MiRNAs represent a great opportunity to enhance the efficacy of radiotherapy treatments—they can be used to profile the radioresistance of tumors before radiotherapy, monitor their response throughout the treatment, thus helping to select intensification strategies, and also to define the final response to therapy along with risks of recurrence or metastatization. Even though many interesting studies support such potential, nowadays most studies on patient data are limited to experiments profiling tumor aggressiveness and response to radiotherapy. Moreover many studies report different although not conflicting results on the miRNAs evaluated for each tumor type. Without doubt, the clinical potential of such molecules for radiotherapy is striking and of high interest.

## 1. Introduction

MicroRNA (miRNA) are small (~22 nucleotides –nt) endogenous, non-coding, single-stranded RNA acting as regulator of gene expression [1]. These molecules negatively regulate gene expression at the post-transcriptional level inhibiting translation of messenger RNA (mRNA), by imperfectly binding within the 3'-untranslated region, and inducing mRNA degradation [2]. Each miRNA act on its target gene by more than one way, depending on the complementarity with the respective mRNA [3]. More than one thousand miRNAs are found in the human genome, each of whom can potentially regulate hundreds of mRNAs, so they play a determinant role in numerous cellular processes: miRNAs were shown to act on around 60% of all protein-coding genes [4,5]. MiRNAs are not the only non-coding(nc)RNA molecules: many other potentially interesting, but still under evaluation RNAs are known, including transferRNA (tRNA), small nucleolarRNA (snoRNA), small nuclearRNA (snRNA), ribosomalRNA (rRNA), small interferingRNA (siRNA), piwi-interactingRNA (piRNA) and long non-codingRNA (lncRNA) [1]; interestingly, the last one has functions similar to miRNAs but is longer (~200 nt) [3]. One reason for interest in miRNA is related to the low demand for technical evaluation. MiRNAs are highly resistant to degradation due to their shortness, thus they are not affected by formalin fixation and can be analyzed using fixed paraffin-embedded samples [6]; moreover their expression levels can be determined in few hours, using a small amount of RNA [2,3]. Dysregulation of miRNA was found associated to different pathologies (e.g., cardiovascular or neurological) [7]; in oncology miRNAs are found to be over- or under-expressed in many tumor types, playing the role of either oncogenes or oncosuppressors [8].

For their biological characteristics and the importance of their roles, miRNAs are proposed as diagnostic biomarkers (being stable, easy to find both circulating in blood, or in urine and stool samples), as aids for subgroup classification in specific malignancies and as prognostic indicators (assessing cancer aggressiveness, trend to metastatization or chemoresistance) [9]. They are also thought of as involved in therapy, through recovery of the dysregulated pattern of expression, or through modification of miRNA expression to enhance the efficacy of therapies [10]. The general strategy for therapeutic use of miRNAs, include two basic approaches: the “*ad hoc*” introduction of molecules mimicking the expression of an underexpressed miRNA that is meant to be restored, or the use of artificial molecules complementary to an overexpressed miRNA whom expression is meant to be decreased. The former approach is achievable by introduction of synthetic double-stranded RNA or vectors expressing the specific pre-miRNA; the latter, either by artificial miRNAs binding the one of interest (antagomiRNAs), or by the so-called miRNA-sponges: molecules presenting multiple binding sites to one or several miRNA [11]. Currently the only attempt at miRNA-driven therapy in patients was for HCV, with satisfactory results [12].

## 2. Role of miRNAs in Response to Ionizing Radiations and Radiotherapy: General Aspects

Radiotherapy (RT), through ionizing radiations (IR), aims to cure tumors determining damages by the production of free radicals at various levels in the neoplastic cell (particularly, but not only on the DNA). Cellular response to IR simultaneously activates a number of signaling pathways mediating the DNA damage response (DDR); failure to repair radio-induced damages leads directly or indirectly to cell death [11]. Complete recovery from these damages affects radiosensitivity: under physiological conditions that avoids the tumorgenesis, while in a clinical setting it determines tumor resistance to RT. MiRNAs are deeply involved in the regulation of DDR processes. This issue promoted a high interest in the potential oncological applications of enhancing efficacy of RT through modification of tumor radiosensitivity. Even though many genes encoding proteins involved in the reactions to IR are known, it is quite difficult to manipulate them: acting by miRNA on those genes and processes is thus of high interest [13]. The same radiobiological background makes the role played by miRNAs in RT treatments also useful to understand and manage treatment-related toxicity. Finally, miRNAs could be useful for monitoring and understanding professional and accidental exposures to IR.

Even though the medical and specifically oncological knowledge about miRNAs is quite well studied, it is still incomplete concerning the frame of interactions involved in radio-sensibility and -resistance, nevertheless the accumulating evidences are promising. Unfortunately, most of the available experiments still do not include the patient’s data, being more based on *in vitro* or xenograft *in vivo* evaluations.

The purpose of this manuscript is to review the most important available experiences on this topic. First we will analyze the evidences of miRNA’s changes of cellular expression levels in response to IR exposure. The specific involvement of miRNAs in the major DDR processes mentioned through the manuscript will be also outlined. Then the evidence of correlation between modification of miRNA levels and radiosensitivity will be described. Finally the main evidence of miRNAs involvement in response to IR and RT by different tumor type and the potential role of miRNAs in issues related to IR exposure other than treatment efficacy will be summarized.

## 3. MiRNA Modifications upon Ionizing Radiations

The roles of miRNAs encompass regulation of DDR, alteration of cell cycle progression, determination of apoptosis and tumor microenvironment changes [14]. Many evidences suggest that miRNA expression is modified under IR exposure, which could indirectly imply their involvement in cellular responses to IR.

An approach to define the miRNAs ruling radiosensitivity-related processes is to determine which ones exhibit significant change of the expression profile after IR exposure [13]. Many experiments have highlighted the correlation between changes in some miRNA profiles and IR exposure [15,16]. Three examples of well-studied miRNAs typically reacting to IR will be reported.

The microRNA-(miR)21 is a promising target of action for future improvement of RT: it controls many biological processes like cellular proliferation, migration, invasion and apoptosis through several pathways, and is overexpressed in most of the more common malignancies [17]. In general, miR-21was shown to be upregulated after irradiation in a variety of tumor and normal cell lines [13].

Shi *et al.*, irradiating mouse hippocampal cells with 0.5 Gray (Gy) of X rays or ^56^Fe ions, stimulated miR-21 expression, whereby miR-21 levels gradually increased after irradiation, without significant differences depending by the type of IR applied [18].

The *let*-7 family represents an interesting but complex group of miRNAs [19]. Acting as tumor suppressors they strongly contribute to the regulation of DDR and proliferation and are underexpressed in many tumors [20]. In general the *let*-7 family regulates the KRAS oncogene (that belongs to the MAPK pathway, regulating protection by IR) [21]. Some studies have revealed that the profiles of expression of *let*-7 miRNAs are modified upon irradiation [13,22,23].

Weidhaas *et al.* reported that after IR exposure of two different cell lines (one lung normal tissue -CLR2741- and one lung tumor -A549-), both reacted to IR showing a downregulation of all but one the miRNA of the *let*-7 family; only the *let*-7g was conversely upregulated [24].

Saleh *et al.* demonstrated that the reduced expression of *let*-7 (-a and -b) miRNAs following irradiation is related to functional p53 that directly binds the a region upstream of the *let*-7 gene, repressing it after irradiation, and to IR-activated ATM signaling upstream of p53 [20,25]. In p53-wild-type mice such a reduction of *let*-7a/b levels was observed in radiosensitive tissues (such as bone marrow) regularly presenting higher levels of *let*-7 miRNAs rather than in radioresistant ones (as in the brain), and correlated with altered expression of proteins in p53-regulated pro-apoptotic signaling pathways. Conversely, decreased expression was not observed in p53 knocked-out mice. Expression of miRNA in response to IR is also complicated by several factors still to be established. For instance, while the modification of the *let*-7 family levels after irradiation was described to be similar for all but one miRNA (*let*-7g), in a report on glioma cells all of them were upregulated upon IR [26].

MiR-34 represents another example of differences in IR-mediated response among different tumor types. It is also targeted by p53 and involved in many processes including cell response to DNA injury, cell cycle progression, cellular proliferation and death: some studies suggest that it is upregulated by IR in several different human tissues [13,27,28,29].

Nevertheless Mert *et al.* reported that miR-34 profiles were modified after IR exposure in HeLa cell culture but not in MCF-7 ones in the same experiment, suggesting that genotoxic stress may be cell-type specific; the same study failed to find a modification of miR-34 expression under exposure to Bleomycin (a chemical agent inducing DNA damage) [30]. That also suggests the presence of a cell- specific behavior in response to radiation injury.

In summary, miRNAs regulate many cellular processes, particularly the ones reacting to IR exposure, as for DDR. The expression of miRNAs changes upon IR, thus they are potential key players determining increased or reduced response to radiotherapy (*i.e.*, radioresistance or radiosensitivity).

## 4. Pathways and Mechanisms of Response to Radiation Damage Regulated by miRNAs

Since miRNAs react to IR, and their activity affects the response to IR by their involvement in the regulatory mechanisms of DDR at different levels and through many pathways, deepening the detail of these steps could allow to more precisely acting on miRNAs to overcome tumor radioresistance.

Briefly, IR injury activates many signaling pathways to promote reaction to the damage (such as PI3K/AKT and MAPK); DNA repair is mediated by activity of damage sensors that can induce a cell cycle arrest at specific check-points; finally some specific repair processes restore the single- or double-strand break (SSB, DSB). We will briefly overview the main processes reporting examples of experiments on the role of miRNAs role [31,32,33].

### 4.1. Signaling Pathways

**Figure 1:**
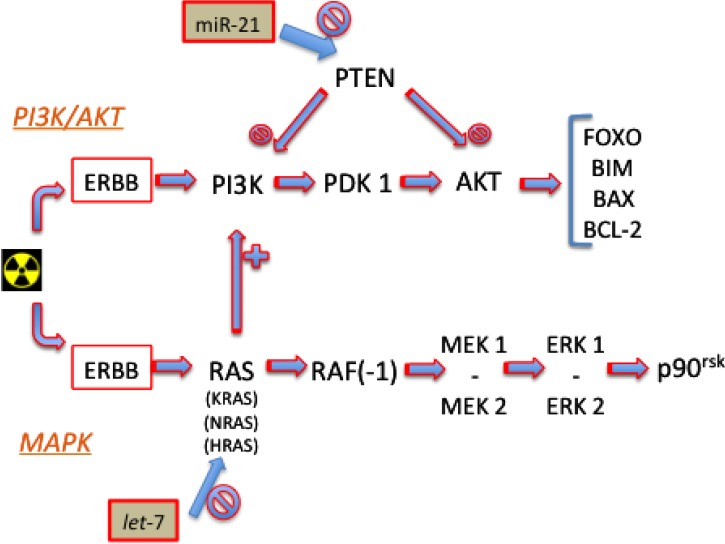
PI3K/AKT and MAPK Signaling Pathways.

Two main pathways are activated by ErbB-family protein activation in response to IR: the phosphoinositide 3-kinase (PI3K)/AKT protein, and the mitogen-activated protein kinase (MAPK), see Figure 1.

#### 4.1.1. PI3K/AKT

In response to IR exposure and damage, the activation of PI3K by ErbB signals leads (through 3'-phosphoinositide-dependent protein kinase 1 –PDK1) to activation of AKT and consequent expression of its downstream effectors (including FOXO proteins, BIM, BAX and Bcl-2). Phosphatase and tensin homolog (PTEN) play a suppressory role in this pathway, limiting the activity of PI3K and AKT.

For instance, miR-21, that reduces activity of PTEN, if overexpressed leads to increased signaling of this pathway through AKT (facilitating DDR and tumor cell survival). Gwak *et al.* showed that blocking miR-21 with anti-miR-21 resulted in radio-sensitization of U373 and U87 cells, whereas overexpression of miR-21 lead to a decrease in radio-sensitivity of LN18 and LN428 cells [34].

#### 4.1.2. MAPK

The activation of the ErbB family can also activate the RAS (from “rat sarcoma proteins”) family (including KRAS, NRAS and HRAS), stimulating a cascade activation to RAF(-1) (from “rapidly accelerated fibrosarcoma”), then mitogen-activated protein kinase kinase (MEK) 1 and 2, then to extracellular signal-regulated kinase (ERK) 1 and 2, and p90^RSK^, along the so-called MAPK pathway. In this pathway a large number of miRNAs are involved playing regulatory roles at different levels [35].

The let-7 family miRNA was found in different studies to target (downregulating) the entire RAS family [21] and, when overexpressed, to radiosensitize tumor cell lines to IR [36].

### 4.2. Cell-Cycle Progression Checkpoints

Three checkpoints in the cell-cycle can represent a stop to arrest in, allowing time for DDR (*i.e.*, G1/S, G2/M and during S). When the damage is recovered, cells re-enter the cycle, while if damage is too broad the cells go on to apoptosis [13]. Levels of cyclins and cyclin-dependent kinase (CDK) proteins regulate cycle progression or arrest: a decrease of their presence contributes to stop the progression at checkpoints. ATM is the one of the main regulators of cycle progression, being (together with ATR, MNR complex and H2AX) a sensor for DNA damage, specifically sensible to DSB detection. Its activation leads, through cell cycle protein kinase (CHK 1 and 2), to either inhibition of CDC25A and consequent cyclins + CDK level reduction, inducing a temporary cycle arrest, or to p53 accumulation (by its phosphorylation) and consequent longer and more stable arrest.

MiR-21was demonstrated to target CDC25A in colon cancer [37], and in general its overexpression can help tumor cells to avoid apoptosis by complete damage repair [31]. On the other hand many miRNAs, particularly including miR-504 [38] and miR-125b [39] regulate the activity of p53 affecting the cycle progression at different levels. Moreover miR-421 was shown to target ATM (by acting the oncogene N-MYC) resulting in S-phase cell cycle checkpoint changes and increased sensitivity to IR through a different complementary manner [40].

### 4.3. Double-Strand Break Repair

When the cell cycle is arrested at a checkpoint specific DDR processes take place [31]. One of the most important DNA damages induced by IR is the double-strand break (DSB): many cellular pathways and repair mechanisms are involved in recovery of DSB, thus determining radioresistance. Two main repair mechanisms are involved: homologous recombination (HR) and non-homologous end-joining (NHEJ). HR is the most efficient, error-free but a time and processing demanding one; it only takes place during phase S and G2 since a template strand is needed. NHEJ is at higher risk of errors but less demanding since the template is not needed: thus it can also be executed apart from checkpoint arrest [33].

#### 4.3.1. Homologous Recombination and Non-Homologous End Joining

Outlining the HR process: different damage sensors (including H2AX and BRCA1) start the process and activate progressively the RAD family (RAD51, RAD52, RAD54), XRCC2 and BRCA2 proteins, finding the proper template in the normal strand, then proceeding to synthesis an damage recovery [31,33].

Moskwa *et al.* showed on culture cells (K562) that antagonizing miR-182 enhances BRCA1 protein levels and protects them from IR-induced cell death, while overexpressing miR-182 reduces BRCA1 protein, impairs HR, and increase sensitivity to IR [41].

NHEJ is activated by the heterodimeric complex XRCC5/XRCC6 that recognizes and binds the end of the DSB, providing framework for a recruited catalytic subunit of DNA-protein kinase (DNA-PKcs), finally activating a complex of XRCC4/Ligase IV that complete the DNA-strand repair.

Yan *et al.* investigated the regulation of HR and NEJR mainly mediated by the aforementioned ATM and the DNA-PK catalytic subunit (DNA-PKcs), respectively [42]. MiR-101 was studied as potential radiosensitizer by inhibition of both those genes. If overexpressed, it reduces the levels of DNA-PKcs and ATM, thus increasing the radiosensitivity of tumor cells. Conversely, inhibition either targeting ATM but not DNAPKcs, or DNA-PKcs but not ATM, only partially reversed the sensitivity of the cells overexpressed with miR-101; finally, combining the two inhibitors almost completely reversed the cell sensitivity.

Also Zheng *et al.*, manipulating both NHEJ and HR by using an artificial miRNA targeting XRCC4 (NHEJ) or XRCC2 (HR) demontrated a clear increase of radiosensitivity [43].

#### 4.3.2. Histone Modifications

miRNAs also influence DSB by action at level of histone modifications and chromatin remodeling needed to let the repairing proteins access the DNA (normally packed in nucleosomes) [13]. Mueller *et al.* analyzed how the miR-99 family modifies radiosensitivity by acting on DDR mechanisms through reduction of DSB repair (like HR or NHEJ) by targeting chromatin remodeling protein complexes [44]. They used two breast cancer lines with different radiation sensitivity (MCF-7 and SK-BR-3) as well as LNCaP and C4-2 cell lines. Cultures were evaluated before and 24 h following treatment with 5 Gy IR. Microarray and clonogenic survival assays were applied to identify different expression levels of miRNA and confirm the radiation sensitivities of the cancer cell lines differentially expressing miR-99 family members. The miR-99 family reduced the efficiency of repair by both HR and NHEJ; moreover they were found to interfere with repair after repeated doses of IR, as used in fractionated RT.

Wang *et al.* showed as miR-138 acts at a different level of the DDR machinery, by targeting a histone variant (H2AX) [45]. In their experiments different cell lines underwent exposure to multiple DNA-damaging agents including drugs (e.g., cisplatin, camptothecin) and IR (with 2 Gy doses), to define the role in regulation of the radiosenitivity presented by different miRNAs. In particular they found that miR-138 downregulates the IR-induced histone H2AX phosphorylation and nuclear foci formation at sites of DNA damage (normally facilitating DNA damage response and repair). Since miR-138 downregulates H2AX, it reduces genomic stability and HR repair, and enhances cellular sensitivity to DNA damaging agents.

In summary, the action of miRNA mediation at several levels of the DDR pathways and processes was explored and confirmed by many experiments, determining a basis for intervention focused on enhancement of RT efficacy.

## 5. Role of miRNAs in Radiosensitivity and Radiosesistance

Theoretically, even though the correlation between exposure to IR and modification of miRNA profiles has been demonstrated, it does not strictly imply their role in regulation of radiosensitivity. That, apart the diagnostic and prognostic purposes, is the most important question for the practical use of miRNAs in clinical practice [13]. It must be highlighted that many factors can be contemporarily involved determining the radiation response, like genetic or epigenetic factors, microenvironment status or blood flow conditions [20], nevertheless enough evidence supports an important contribute by miRNA interactions. Many studies have explored which miRNAs are more involved in processes related to radiosensitivity [46].

### 5.1. Experiments on Global miRNA Expression

At least two experiments analyzed the involvement of miRNA in tumor radiosensitivity from a global point of view. The main regulators of miRNA biogenesis are the ribonucleases DROSHA, DICER, ARGONAUTE-2 (Ago2) and EXPORTIN-5 (Exp5) by direct inactivation of which the changes of miRNA expression and its rebound on response to IR were analyzed.

Briefly, miRNAs are initially transcribed by RNA polymerase II as large precursors (pri-miRNA), then processed by a ribonuclease (as DROSHA) to a second precursor (pre-miRNA) that is carried to cytoplasm by Exp-5, to be then processed by another ribonuclease (DICER) to be active [3].

Kaemer *et al.*, downregulating Ago2 or DICER proteins by using RNAi in lines of immortalized and primary endothelial cells, found that the global suppression of miRNA expression led to increased cell death after γ-irradiation with 2.5 Gy, indicating a prosurvival and anti-apoptotic function of miRNAs [47]. The authors also identified that the lack of miRNAs caused by such suppression only affected cell cycle checkpoint activation and apoptosis, while DNA double-strand break repair remained normal, suggesting an independent activation of the two response pathways rather than a concerted DNA damage response.

That report is significant, for the purpose of this review, to highlight the evidence of global correspondence between miRNA expression levels and radiosensitivity. Nevertheless it should be stressed that many other correlations are implied in radiosentization, even within mechanisms related to the mentioned ribonucleases, making it difficult to completely understand the process.

Surova *et al.*, for instance, in another similar study on human non-small and small cell lung carcinoma cell lines had different results [48]. They found that DROSHA and DICER were expressed at higher levels in radioresistant, but not in sensitive cell lines. Cells were exposed to γ-irradiation at a dose of 8 Gy using a ^60^Co source and analyzed at different time intervals. They down-regulated either DICER or DROSHA by using siRNAs targeting human DICER1, DROSHA and Ago2, and non- targeting siRNA as a negative control. That manipulation failed to gain effect on the sensitivity of cells to irradiation.

On the other hand, Francia *et al.* demonstrated in experiments on human, mouse and zebrafish cell lines that DROSHA and DICER are involved in DNA repair by the production of small RNA molecules other than miRNA, suggesting also other different pathways of interaction [49]. The DDR foci (following different sources of DNA damage, including a single DSB at a defined and traceable genomic locus) were decreased in DROSHA and DICER knocked-down cell lines, due to lacking of site-specific DICER- and DROSHA-dependent small RNAs, named DDRNAs. In summary authors demonstrated the activation through DICER- and DROSHA-dependent RNA products acting differently from canonical microRNA proteins in response to various sources of DNA damage (not only IR). These evidences suggest a very complex pattern of cellular interactions upon IR, in any case including miRNAs as regulators.

### 5.2. Experiments on Specific miRNA Expression

Many experiments focused on demonstrating the role played by specific miRNAs on radiosensitivity. The previously mentioned paper by Weidhaas *et al.* (see Section 3) not only showed a correlation between *let*-7 family expression levels and IR exposure tumor cells lines (A549), but also how the manipulation of such a protein’s expression levels significantly impacts radiosensitivity [24]. The cells were transfected with synthetic pre-*let*-7 molecules or control pre-miRNA containing scrambled sequences, and significant radiosensitization was found in cells treated with pre-*let*-7b or pre-*let*-7a compared with control. The opposite was found with the anti-let-7g whose overexpression was associated to radioresistance. They also reproduced the experiment *in vivo* (in the nematode *C. elegans*).

Wagner-Eicker *et al.* investigated with microarrays 361 miRNAs profiles in response to 2 Gy photon-irradiation in primary human dermal microvascular endothelial cells (HDMEC) [50]. To define the role of each specific miRNA determining the radioresponse, cells were transfected with miRNA precursor or inhibitor. The authors showed that cellular radiosensitivity was significantly modified by differential expression of some miRNA in particular: miR-125a and miR-189 had a radioprotective effect, while miR-127 and *let*-7g enhanced HDMEC radiosensitivity.

In summary, many experiments have demonstrated that altered miRNA expression affects cellular radiosensitivity. Sensibility or resistance to RT induced by an increase or reduction of miRNA levels can depend on cell type, specific miRNA up- or down-regulated and pathway triggered or silenced by downstream effects. Targeting specific miRNAs could enhance radiosensitivity or limit radioresistance, thus producing more relevant clinical effects in response to RT.

## 6. Role of miRNAs in Tumor Type Subsettings

Many evidences have confirmed and deepened the correlation between miRNAs and cancer [8,10]: the importance of diagnostic application of miRNA is wide and promising [51] and allows one to determine which miRNA is more involved in different tumor types [2,4,52]. Determination of patterns of tumor aggressiveness, trend to metastization and resistance to chemotherapy were also evaluated in different cancer subgroups [9,53].

A relatively lower, albeit wide number of studies have focused on the roles of miRNAs in response to IR in different tumor types. Experiments are reported both on cell line cultures or in *in vivo* models, and few of them also include data from patient specimen analyses [20].

The main evidences available from literature about the role and impact of miRNA expression on radiosensitivity for different tumor types that usually require RT as key component of treatment approach are overviewed. A summary of the detailed evidences is reported in Table 1.

**Table 1:** Correlation of miRNA expression levels changes and Radiosensibility Modifications in Different Tumor Types.

Tumor Type	MiRNA Expression	Radiosensibility Modification
Prostate	↑ miR-521	↑
	↑ miR-106b	↓
Pancreas	↑ *let*-7	↑
	↑ miR-34	↑
	↑ miR-23	↓
	↑ miR-99m	↑
Esophageal	↑ miR-31	↑
	↑ miR-22	↑
	↓ miR-21	↑
	↓ miR-301a	↓
	↓ miR-141	↓
	↓ miR-18b	↓
Lung	↑ *let*-7	↑
	↑ miR-449a	↑
	↑ miR-27a	↑
	↑ miR-101	↑
	↓ miR-155	↑
	↑ miR-210	↓
	↑ miR-34	↑
	↓ miR-21	↑
Rectum	↑ miR-145	↑
	↑ miR-622	↑
	↑ miR-630	↑
	↑ *let*-7	↑
	↑ miR-196b	↑
	↑ miR-450a-b	↑
	↑ miR-99	↑
	↑ miR-215	↓
	↑ miR-190b	↓
	↑ miR-29b2	↓

↑: increase; ↓: reduction

### 6.1. MiRNAs in Prostate Cancer

The role of miRNAs in prostate cancer has been widely evaluated as a diagnostic, prognostic marker and also as indicator of response to hormone- and chemo-therapy [20,54,55,56] but the role in response to IR is less well studied and mostly confined to studies on cell cultures.

Leung *et al.* [57] exposed prostate cancer cell lines (PC3) to different dose levels (0,2,6,10,14 and 18 Gy). The authors identified six miRNAs whose expression increased after irradiation (miR-9, miR-22, miR-25, miR-30a, miR-550a, miR-548h) and 16 that decreased upon IR (*let*-7c/d/e, miR-15a, miR-17, miR-30d, miR-92a, miR-125a, miR-197, miR-221, miR-320b, miR-342, miR-361, miR-374a, miR-501, miR-671).

Josson *et al.* reported significant changes in miR-521 and miR-34c levels after photon beam irradiation of prostate cells with 6 Gy [29]. MiR-521 in particular was demonstrated in the same experiment to be able to modify sensibility to IR (and thus to RT): its overexpression sensitizes to RT while its ectopic inhibition determines radiation resistance of prostate cells.

Li *et al.*, screening 132 miRNAs in prostate tumor cells (LNCaP) in response to IR, showed a significant change of expression levels of miR-106b [58]. Artificial overexpression of miR-106b was associated to significantly lower radiation-induced growth inhibition, suggesting it as a potential target for overexpressing tumors undergoing to RT.

It should be highlighted that the last two studies reported about two miRNAs that were not highlighted by the other one, suggesting that many other potential targets have to be detected. Morever many other influencing and otherwise confusing factors must be elucidated.

John-Aryankalayil *et al.*, for instance, took into account the issue of dose fractionation. That is of high clinical potential interest, since most of the published studies refer to single exposures of IR on cell cultures. They studied different prostate cell lines (LNCaP, PC3, DU145) under either single exposure or fractionated irradiation [59]. Irradiation was administered to a total dose of 5 and 10 Gy. Fractionated schedule was represented by 0.5 Gy per 10 times (twice a day), or 1 Gy per 10 times (twice a day). In their experiment the fractionation of dose altered miRNAs more than the single exposure, and some tumor suppressors (including miR-34 and *let*-7) were upregulated by dose fractionation in radiosensitive cell lines.

### 6.2. MiRNAs in Pancreatic Cancer

MiRNAs aiding to define diagnosis and prognosis related to pancreatic tumor are under deep evaluation [60,61]. Oncogenic signatures were proposed: overexpression of miR-155, miR-203, miR-210 and miR-222 was significantly related to poorer overall survival in a study on a cohort of 56 pancreatic ductal adenocarcinomas (PDAC) [62]. Nevertheless less extensive analysis is still related to their radiosensitizer role [20].

Oh *et al.* published an experiment on the role of Lin28-*let*7 in modulating radiosensitivity on human tumor cells from pancreatic (ASPC1) and lung (A549) cultures [36]. Inhibiting Lin28, a repressor of *let*-7, radiosensitized ASPC1 (and A549, lung tumor) cells by attenuation of K-Ras expression.

Ji *et al.* analyzed the role of miR-34 in human pancreatic cancer cell lines (MiaPaCa2 and BxPC3) [63]. Restoration of miR-34 expression in cancer cells downregulated Bcl-2 and Notch1/2 pathways, significantly sensitized cells to photon-irradiation and to chemotherapy, inhibited clonogenic cell growth and invasion, and induced apoptosis and G1 and G2/M arrest in cell cycle.

Wang *et al.* evaluated the contribute and mechanism of radioresistance of miR-23 through action on authophagy [64]. Authophagy represents a cellular process of defense by IR-mediated damage, that provide and contribute to radioresistance [65]. On radioresistant cell lines of human pancreatic tumor (BxPC3 and PANC-1) they defined as miR-23 was underexpressed in both cell lines. Analysis of changes after irradiation included the evaluation of D0 (*i.e.*, dose required to reduce survival to 37% of its value), the surviving fraction at 2 Gy, and the sensitization enhancement ratio at 10% [66]. They demonstrated that resistant pancreatic cells show underexpression of miR-23, and increased autophagy, conversely, overexpression of miR-23 reduced radiation-induced autophagy, sensitizing pancreatic cells to IR.

Wei *et al.* studied miR-99m as regulator of radiosensitivity acting on a different pathway in a very interesting *in vitro*/*in-vivo* experiment strongly suggesting for clinical application in RT [67]. The kinase known as mammalian target of rapamycin (mTOR) is a target of PI3K/Akt pathway and plays a crucial function in cell-growth regulation, proliferation and survival [68]. When mTOR is overexpressed, it contributes to tumor progression and drug-resistance [69]. mTOR activation and overexpression is correlated to higher resistance to RT in pancreatic tumors. miR-99m, when overexpressed, can dowregulate mTOR by direct targeting, thus potentially increasing RT efficacy. *In vitro* (on PANC-1, Capan-2 and BxPC-3 cell lines), and *in vivo*, they tested the combination of IR and an mTOR inhibitor. The mTOR inhibitor AZD8055 was administered together with IR in single doses of 1, 2.5, 5 or 10 Gy. *In vitro* cell growth inhibition and apoptosis was synergistically promoted. Moreover, they treated human pancreatic cancer xenografts in mice, with fractionated IR (*i.e.*, 2Gy four times) alone, or with AZD8055 treatment, or both combined. The combined treatment enhanced the therapeutic effect – tumor volume was shrank to 278 mm^3^ after combination treatment for 3 weeks compared with single radiation (678 mm^3^) or AZD8055 (708 mm^3^) treatment (*p* < 0.01). The mentioned evidences are complementary but focus on different miRNAs and pathways of action, requiring further evidences and a global definition of this issue [70].

### 6.3. MiRNAs in Esophageal Cancer

Clinical significance in term of diagnosis, prognosis and profiling of modified expression is well studied in this subgroup [71,72], moreover the correlation of miRNA profiles and chemotherapy response is under evaluation [73,74]. Growing interest is focused on the potential role of miRNAs in RT as modifiers of radiation response.

Zheng *et al.* individuated a sequence of 10 miRNAs upregulated and 25 downregulated ones in a culture of esophageal tumor cells with induced radioresistance after repeated irradiation compared to the parental cell line (KYSE-150R) [75].

On the same radioresistant cell line (KYSE-150R) Su *et al.* performed a similar, more recent experiment [76]. They confirmed the statistical significance of downregulation in hsa-miR-301a, hsa-miR-141 and hsa-miR-18b expression between the resistant and parental cell lines.

A recent study by Huang *et al.* also determined the implication of miR-21 on inducing resistance to IR [77]. Analyzing esophageal squamous cancer cells culture (TE-R60) they showed an increment of the radiosensitivity by inhibiting miR-21.

Two recent studies seem quite promising for their clinical implications. Lynam-Lennon *et al.* addressed an *in vitro*/*in-vivo* experience [78]. In the *in- vitro* part of the experiment, using radioresistant and parental esophageal cell lines (OE33 R and OE33 P), they demonstrated that miR-31 levels were significantly differently expressed among the cell lines, both at basal level and in response to IR (after 2 Gy exposure). Moreover, artificial increase of miR-31 levels in resistant cells (by transfection of Pre-miR-31) significantly (*p* < 0.05) sensitizes to radiation compared with control. Intriguingly, in the *in vivo* part of the study, they also evaluated the expression and correlation of miR-31 profiles in tumor tissue biopsies with clinical outcome of a group of 19 patients who underwent to radiochemotherapy (to a dose of 40.5 Gy, 2.67 Gy per fraction, plus cisplatin and 5-fluoruracil) and following surgery. Clinical outcome was defined in terms of tumor regression grade (TRG): a pathological score of the response to preoperative treatment in five levels, from the pathological complete response (pCR; *i.e.*, TRG1) to the absence of response (TRG5). The baseline expression of miR-31 was significantly higher in good responder (TRG1-2) patients than in poor ones (TRG4-5) [*p* = 0.0279], moreover patients achieving pCR had a significant higher rate of miR-31 than TRG5 ones [*p* < 0.05]. Considering the demonstrated *in- vitro* effect of restoration of IR sensitivity by introduction of miR-31, it makes this appealing for clinical purposes.

Wang *et al.* recently published a study correlating miR-22 profiles to clinical outcomes [79], using tissue samples of esophageal squamous cell carcinoma (ESCC) and surrounding normal tissue from 100 patients, plus culture cell lines (EC9706, KYSE510, KYSE450, KYSE150). Tests on biopsies showed a significant difference in expression of miR-22 between tumor and normal tissue in specimens. Moreover the proliferative ability was increased in cells at low miRNA-22 level and decreased in ones at high-expression of miRNA-22. All 100 patients underwent surgery, and 58/100 received postoperative radiochemotherapy (to a dose of 50 Gy, 2 Gy per fraction, plus cisplatin and 5-fluoruracil). No correlation was found between miRNA-22 expression and overall survival (*p* = 0.237). An interesting difference was found for the subset of patients who underwent to radiochemotherapy: the survival rate of miRNA-22 high-expression patients was higher than that of miRNA-22 low-expression patients (*p* = 0.042), while such difference was not revealed among patient who only received surgery. That supports the implication of miR-22 in determining a clinical effect in response to RT and sustains the interest for clinical approaches in such direction.

### 6.4. MiRNAs in Lung Cancer

The role of miRNAs in lung cancer has been extensively studied and their promising diagnostic and prognostic potential is deeply studied [80,81]. Some studies also focused on the regulation of radiosensitivity managed by miRNAs and its clinical implications for RT treatments, most of them driving experiments on cell cultures only. If the previously mentioned paper of Surova *et al.* [48] suggested that a global manipulation on the ribonuclease complex was not able to modify lung tumor radiosensitivity (see Section 5.1), other studies investigated specific miRNAs.

Two papers profiled miRNA signatures of radio-resistance and -sensitivity for lung cancer. Shi *et al.* [82], determined on a culture of human lung carcinoma cell line (A549), a signature of eight miRNAs both responding to 20 and 40 Gy (with ^137^Cs γ-ray source) including: miR-345, miR-885-3p, miR-206, miR-516a-5p, miR-16-2, miR-106a, miR-48c-3p and miR-127-3p.

Wang *et al.* published one of the few studies that used patient specimen data, defining a signature of the radio-sensitive and -resistant miRNAs from a group of 30 non-small cell lung cancer patients undergoing postoperative RT on the basis of overall survival and local or distant recurrence rates [83]. They defined a group of 12 miRNAs associated to the sensitive outcome profile, five of whom significantly upregulated (miR-126, miR-let-7a, miR-495, miR-451 and miR-128b) while seven were downregulated (miR-130a, miR-106b, miR-19b, miR-22, miR-15b, miR-17-5p and miR-21) compared to the IR resistant group.

Other studies have focused on specific miRNAs and pathways of interaction with radiosensitivity. Weidhaas *et al.* [24], Oh *et al.* [36] and Arora *et al.* [84] confirmed the radiosensibilization associated to overexpression of the *let*-7 family (through the K-Ras pathway).

Liu *et al.* demonstrated in two lung cancer cell lines (CL1-0 and CL1-5) upon irradiation (at 0, 2, 5 and 10 Gy) that the overexpression of miR-449a was associated to increased radiosensibilization due to DNA damage, apoptosis and altered cell cycle distribution [85]. Similar results were found by Di Francesco *et al.* for miR-27a (in A549 cultures) after 2 Gy γ-irradiation exposure [86].

Chen *et al.*, studying miR-101 in NSCLC cell lines, focused on a particular point [87]. Ectopic miR-101 was able to radiosensitize most NSCLC cells, except for the NSCLC cell lines that had a much higher endogenous miR-101 level. Their paper suggests that the use of miRNA as a therapeutic tool also requires an adequate attention to the baseline endogenous level of the miRNA.

At least two papers analyzed the role of miRNA in the hypoxia-mediated resistance to IR and the potential therapeutic role to increase radiosensitivity through this way. Barbar *et al.* studied miR-155 in tumor cell lines (A549 and H460) showing that its inhibition radiosensitize hypoxic cells [88]. Grosso *et al.* indicated that overexpression of miR-210 increases resistance to IR under hypoxia (evaluated by HIF-1) [89].

Regarding lung cancer, miR-34 is one of the more interesting miRNAs regulating cellular sensitivity to radiation. At least three studies have confirmed its importance:

Duan *et al.*, (on culture cells A549 and H1299, with or without exposure to γ-irradiation) [90], and Balça-Silva *et al.* (on p53-wild-type, KRAS mutated culture lung cancer cells A549) [91] showed that overexpression of miR-34 enhances tumor response to radiation. Similar results were also confirmed by Kang *et al.*, who also highlighted the action of miR-34a in suppressing the Notch-1 signaling expression (a pathway involved in critical cell fate decisions by modulating cell proliferation) in an *in vitro*/*in-vivo* study [92]. In their experiment they confirmed the role of miR-34a inhibiting that pathway and also the potential role of two flavonoid compounds (rhamnetin and cirsiliol) regulating Notch-1 to target it and thus increase radiosensitivity both in NSCLC cells with different levels of radioresistance (NCI-H1299 and NCI-H460) and in xenografts. In the xenograft model, the tumor volume was significantly reduced by combinational treatment with irradiation and the two drugs compared with irradiation alone.

A final promising miRNA that modulates radiosensibility of lung cancer models is miR-21. The role of miR-21 in suppression of PTEN activity, determining an oncogenic effect, was confirmed by Liu *et al.* on cell cultures (A549) [93]. The induced downregulation of miR-21 significantly inhibited tumor cells’ growth, migration and invasion, and reversed radioresistance.

Wang and colleagues focused on miR-21 an analysis both based on patient tumor specimens and on lung tumor cell (A549) culture [94]. Their evaluation on cell lines confirmed the radioprotective effect of overexpressed miR-21: silencing miR-21, after IR exposition (to 0, 2, 4, 6 and 8 Gy) an inhibition of cell growth, increased cell cycle arrest in G1 and increased apoptosis rate were observed. They evaluated samples from 60 patients including tumor and surrounding normal tissue. The tumor expression of miR-21 was significantly higher than normal tissue (*p* = 0.0001), moreover higher miR-21 levels were associated to lower survival rates (*p* = 0.007), lymph-node invasion (*p* = 0.015) and clinical stage (*p* = 0.004) being the miRNA level an independent prognostic factor at multivariate analysis. We can summarize that the most promising miRNAs for clinical applications in RT for lung cancer seem to be the *let*-7 family, miR-34 and miR-21.

### 6.5. MiRNAs in Rectal Cancer

Rectal cancer has been less extensively studied for miRNA implications than other tumor types, but is of high interest for RT treatment, being a crucial element of multidisciplinary approach. Four interesting studies have evaluated the prognostic profiling by miRNA of clinical response to preoperative RT, on the basis of patient data:

Drebber *et al.* analyzed specimens from 40 patients (T3/4/Nx) for expression of miR-21, miR-143 and miR-145 (on the basis of their overexpression in colorectal carcinoma), testing it before and after treatment consisting of radiotherapy (50.4 Gy) plus 5-fluoruracil [95]. Therapy response was assessed according to pathological tumor regression. They found significant correlation between miR-145 expression and tumor regression. Patients with a low intratumoral post-therapeutic expression had significantly more often a worse response to neoadjuvant therapy compared to patients with a high expression of miR145.

Della Vittoria Scapati *et al.*, determined the association between miRNA expression (on untreated tissue from biopsy) and tumor regression grade (TRG) on 38 T3/4/Node-positive patients who underwent to RT (45 Gy) plus concomitant capecitabine and oxaliplatin, in terms of achievement of pCR (*i.e.*, TRG1) *vs.* not-pCR (TRG > 1) [96]. They found that 11 miRNAs (miR-1183, miR-483-5p, miR-622, miR-125a-3p, miR-1224-5p, miR-188-5p, miR-1471, miR-671-5p, miR-1909, miR-630, miR-765) were significantly overexpressed in TRG1 patients, while two (miR-1274b, miR-720) were underexpressed. In particular, miR-622 and miR-630 had a 100% sensitivity and specificity in selecting TRG1 cases.

Svodoba and co-workers profiled miRNA expression levels of 20 patients undergoing to RT (45+5.6 Gy) plus capecitbine or 5-fluoruracil (on untreated tissue from biopsy) [97]. Patients were separated on the basis of treatment responses into “responders” (*i.e.*, achieving TRG1 or 2), or “non-responders” (*i.e.*, TRG3 or 4). With significant difference: miR-215, miR-190b and miR-29b-2 for “non-responders”, and let-7e, miR-196b, miR-450a, miR-450b-5p and miR-99a for “responders” were respectively overexpressed. Interestingly, on the basis of such elements, nine of 10 responders and nine of 10 non-responders (*p* < 0.05) have been correctly classified.

Kheirelseid and colleagues also defined a specific signature predictive for complete *versus* incomplete response to RT on 12 specimens from patients’ biopsies: three miRNA transcripts (miR-16, miR-590-5p and miR-153) predicted complete *versus* incomplete response and two miRNA transcript (miR-519c-3p and miR-561) predicted good *versus* poor response with a median accuracy of 100%.

Globally summarizing, many efforts have been done to profile several tumor types for the miRNA associated to radioresistance and determine potential site(s) of action to overcome it. Few studies (as for esophageal, lung and rectal cancers) evaluated the data based on patients’ specimens. Moreover some evidence suggests an even more complex framework due to the potentially different miRNA expression profiles in response to different radiotherapy delivery modalities, like either conventional or intensity-modulated (IMRT) radiotherapy (IMRT), for the same dose [98].

## 7. Implications of miRNAs in Other Radiotherapy-Related Issues

The potential clinical roles of miRNA also involve issues other than increased treatment efficacy:

### 7.1. Radiotherapy-Related Toxicity

Very few but highly interesting reports suggest the potential use of miRNAs for early detection or reduction of RT-induced toxicity. Being RT a loco-regional treatment, its toxicity is mostly confined to the site of irradiation (apart from the role played by systemic concomitant chemosensibilization). Often the treatment related toxicity requires interruptions of therapy with a potential detriment of results; on the other hand toxicity due to RT could be reflected by quality-of-life threatening sequelae. Being able to overcome these side effects, or to interpret their signs before an irreversible instauration could lead to an amelioration of patient’s comfort and better global result and was addressed in many attempts [99,100]. Talwar *et al.* showed in mice that transcriptional factors including RNA-binding proteins (RBPs) and miRNAs can affect mRNA regulation and apoptosis in oral mucositis, and how by acting on these processes a potential benefit at the oral epithelial compartment is expected [101]. Moreover, Hamama *et al.* reported about the potential role of miR-210 in regulating radiation-induced intestinal fibrosis: after validation in a cellular model, they showed its overexpression in rectal patient’s samples removed at surgery after radiotherapy. They also demonstrated repression of miR-210 by anti-fibrotic therapy [102].

### 7.2. Professional and Accidental Exposure to IR

Professional exposure to low doses of IR, or accidental ones to low-to-high doses (as in nuclear incidents) were widely evaluated and many strategies were suggested for early detection of sign of risk of IR damage or secondary cancers; in this context miRNAs can help to define such situations with high precision [103,104] and potentially offer a specific, selective way to recover.

## 8. Conclusions

MiRNAs represent a great opportunity to enhance RT treatment efficacy: they can profile the radioresistance of tumors before treatment delivery, monitor the response through the treatment, thus helping to select intensification strategies, and also to define the final response to therapy along with risks of recurrence or metastatization. Even though many interesting studies support such a potential, nowadays most of the experiments using patient data are still limited to aggressiveness and response to radiotherapy profiling. Moreover, many studies report different although complementary results for the evaluated miRNAs for same tumor type. Nevertheless the clinical potential of such molecules for radiotherapy is striking.
